# Assessment on Changes of Ecosystem Carbon Storage in Reservoir Area due to Hydroproject

**DOI:** 10.1155/2022/7511216

**Published:** 2022-01-27

**Authors:** Shan Long, Shenbei Zhou

**Affiliations:** ^1^Management School, Hunan City University, Yiyang 413000, China; ^2^Business School, Hohai University, Nanjing 211100, China; ^3^Water Resources Economics Research Institute, Hohai University, Nanjing 211100, China; ^4^Eastern Natural Resources, Environment and Sustainable Development Research Center, Nanjing 210098, China

## Abstract

Hydropower offers significant value for global carbon peak and carbon neutrality. However, the construction of hydropower stations leads to significant changes in land use and cover structure in reservoir areas, which affect ecosystem services including carbon balance. Furthermore, the development and operation of hydropower project require vast investment. However, the reservoir ecosystem's carbon storage and carbon emission reduction caused by hydropower could offer economic benefits when the official carbon market trading in China was launched in 2021. Therefore, it is necessary to assess comprehensively the changes in carbon storage and its value to the ecosystem in reservoir areas. The evaluation is of great importance for carbon loss reduction, land management, and hydropower development. This study provides a comprehensive and effective framework for evaluating changes in carbon storage and has its value to the reservoir ecosystem. It combines land utilization classification data obtained from remote sensing image interpretation and the Integrated Valuation of Ecosystem Services and Tradeoffs (InVEST) carbon storage model. Based on the case study of the Xiluodu reservoir area, they were evaluated from two aspects: physical quantity and value quantity. The results show that the carbon storage in the Xiluodu reservoir area increased by 8,504.42 Mg from 2000 to 2018. The spatial distribution of the carbon storage shows a trend of high in the north and west, but low in the south and east. The construction of hydropower stations and the rise of reservoir water level covered a large amount of land, which led to the loss of carbon storage in reservoir areas. By implementing soil and water conservation and vegetation protection policies, parts of the cultivated land and grassland were converted into forestland, which was the main source for increasing the ecosystem's carbon storage. Moreover, carbon emission reduction was achieved by hydropower. In terms of the monetary value, the carbon storage value of the reservoir ecosystem increased to 19 million RMB during the construction period (2005–2015). The carbon storage value of the reservoir ecosystem increased to 611 million RMB during the operation period (2015–2018). The latter was greater than the maintenance cost of the hydropower station and exceeded the amortized cost of hydropower development, indicating the feasibility and economic benefits of hydropower development. These findings provide guidance for future hydropower development decisions in Jinsha River Basin and also others.

## 1. Introduction

Global warming is one of the biggest challenges to sustainable development of human society and natural ecosystem. It has gradually become a hot issue worldwide [1–3]. Previous studies show that carbon emissions caused by human activities are the main reason of climate change, mainly from burning of fossil fuels [2]. China is working on local and global environmental challenges [4]. These include a commitment to peak CO_2_ emissions around 2030 according to the Paris Agreement signed in 2015 and a commitment to achieve carbon neutrality by 2060 at the 75th UN General Assembly in 2020 based on the Paris Agreement [5–7]. Considering the global carbon peak and carbon neutrality deadlines, it is necessary to reduce dependence on fossil fuels, attach importance to clean energy development, and optimize energy structure.

As the largest share of clean energy (85%) and the most mature and sustainable power resource, hydropower continues to be the focus of future development [7, 8]. However, extensive hydropower development profoundly impacts the ecosystem, changing the local land use status, ecological diversity, and vegetation coverage [9, 10]. Previous studies showed that land use and cover change (LUCC) is the main cause of global carbon cycle imbalance and an important source of carbon emissions that ranks secondary after fossil fuels [11]. The impact of LUCC on the carbon cycle has become an important aspect of ecological benefit literature, mainly focusing on impacts of soil carbon storage and biomass carbon storage in specific ecosystems. However, the impact of LUCC on multiple carbon pools in composite ecosystems remains unclear [12].

Previous studies have confirmed the influence of hydropower station construction on changes of land use and cover type in the reservoir area. Zhang et al. found that the cultivated land, forestland, and grassland in the reservoir area kept decreasing, while the built-up area and the water area kept increasing during 1978–2005 due to the construction of the Three Gorges Dam and environmental protection policies after the completion of the dam [13]. Guan et al. believed that the construction of the Three Gorges Dam changed the land use structure. They used Logistic-CA-Markov and WLC-CA-Markov models to simulate Chongqing's future land use pattern in the Three Gorges Reservoir area. It was found that the grassland and cultivated land in the reservoir area would continue to decrease while the water area would remain stable, and the forestland and construction land would continue to increase [14]. Rufin et al. collected 178 cases of land use structure change caused by hydropower station construction and concluded that land cover change is mainly related to hydropower stations. Moreover, scholars have discussed the influence of hydropower station construction on soil carbon storage and forest carbon cycle in the reservoir area [15]. Kumar and Sharma and Pereira et al. studied the influence of water flooding of hydropower station on the soil carbon content in nearby forests [16, 17]. Dullah et al. discussed the influence of hydropower station construction on forest plant carbon. They believed that the carbon sequestration rate of the remaining forest in studied areas was still considerable, and the carbon sequestration potential was acceptable [18].

However, previous studies on influence of hydropower station construction on carbon storage were mainly concentrated on one or two carbon pools (plant carbon and soil carbon) of particular ecosystem. Therefore, it is necessary to comprehensively evaluate changes in various carbon pools in the reservoir area. Such an assessment is very important for carbon reduction, land management of future reservoir areas, and hydropower station management. The Integrated Valuation of Ecosystem Services and Tradeoffs (InVEST) model can be linked to Geographic Information Systems (GIS) mapping to evaluate various service functions of ecosystem services [19–21]. The carbon storage and capture models in the InVEST model include four carbon pools: aboveground biomass, underground biomass, dead organic carbon, and soil carbon. They can accurately reflect changes in carbon storage caused by LUCC in terrestrial ecosystem.

The InVEST model has been widely used to evaluate and simulate carbon cycles of terrestrial ecosystems at different scales due to its visualization on evaluation results, data accessibility, and ease of operation [22–24]. However, previous studies with the InVEST model mainly focused on impacts of urban expansion, shelterbelt construction, and returning farmland to grassland on carbon storage. Few scholars used the InVEST model to study impacts of land use change caused by hydropower station construction on carbon storage in terrestrial ecosystems. Moreover, the development and operation of hydropower require vast investment. Taking the hydropower base in the Jinsha River Basin as an example, over 500 billion RMB has been invested in hydropower during past 20 years. With the official launch of carbon market trading in 2021, changes on carbon storage in the reservoir ecosystem and carbon emission reduction caused by hydropower during operation also have specific economic values. Therefore, this paper estimates the value of changes in ecosystems' carbon storage in the reservoir area from the perspective of carbon market value, considering carbon emission reduction caused by hydropower. Then, the value is compared with the hydropower investment and operating costs to evaluate the economic contribution to the reservoir area in different periods. The study provides guidance for decision-making of hydropower development and reservoir ecosystem management. Moreover, the study provides a comprehensive and effective framework for evaluating changes in carbon storage to reservoir ecosystems and their economic value which is based on integrating land use classification data obtained from remote sensing image interpretation and the InVEST carbon storage model.

The Jinsha River Basin has the largest hydropower resources in China. At present, three of the world's top ten hydropower stations are located here in terms of installed capacity and adequate resources for subsequent development. The Xiluodu hydropower station is the fourth largest station in the world and is located in the Jinsha River Basin. In the past 20 years, a large amount of land resources has been occupied by the construction, infrastructure, and immigrant resettlement along with the station development. The stored water has flooded the adjacent land causing the land use and cover of the reservoir area to change significantly. Functions of ecosystem service in the reservoir area have been affected, including carbon balance. Therefore, the Xiluodu reservoir area was selected for this study as a typical case. Particularly, four stages were selected: the period before hydropower station was built (2000); the beginning of construction (2005); the completion of construction (2015); the operation period (2018). Changes in carbon storage in the reservoir area from 2000 to 2018 were studied with an in-depth discussion on impacts of the station. Detailed topics show as follows:

1. Distribution of land use and cover types in Xiluodu reservoir area in 2000, 2005, 2015, and 2018 due to hydropower station construction, combined with satellite data and environmental changes.
2. Current changes of carbon storage in reservoir ecosystem and its main reasons, combined with land use, cover types, and carbon density data.
3. Calculating economic value associated with carbon storage changes in the reservoir area with the market value method. The method accounts for carbon emission reduction caused by hydropower and compares the hydropower investment and operation costs to evaluate the economics of hydropower development.

## 2. Materials and Methods

### 2.1. Research Area

**Figure 1:**
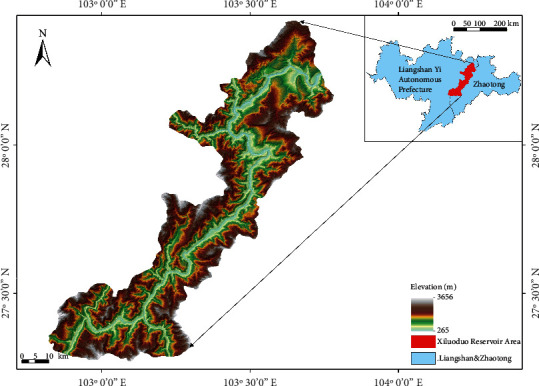
Geographic location of Xiluodu reservoir area.

The reservoir area is located at 102°49′36″E-103°48′9″E, 27°15′20″N-28°17′31″N (Figure 1). The reservoir area (3,613,657 hm^2^) is situated in a small watershed within the first ridgeline between the Baihetan hydropower station and the Xiluodu dam site. This location is within the first level watershed of Xiluodu hydropower station which is at the junction of Yunnan and Sichuan Province. There are nine counties (districts) in these two provinces. This area belongs to subtropical monsoon climate which is dry and hot. The annual precipitation ranges from 600 mm to 1100 mm. The annual average temperature varies from 10.1°C to 19.7°C. The altitude ranges from 265 to 3,656 m (data source: Environmental Impact Assessment Report of Xiluodu Hydroproject, Edited by Chengdu Engineering Corporation Limited, April 2005). The reservoir is an ecologically sensitive area in China with steep slopes, high and middle gorge landforms.

The Xiluodu hydropower station is located in the Xiluodu Valley because of abundant water in this area which borders Yongshan County of Yunnan Province and Leibo County of Sichuan Province at the lower Jinsha River. The main program of the hydropower station was started in June 2004 and put into operation in October 2015. After Xiluodu hydropower station started operation, the water area in the reservoir area increased by approximately 10,000 hectares, and the cultivated land decreased by approximately 8,000 hectares.

### 2.2. Research Framework

**Figure 2:**
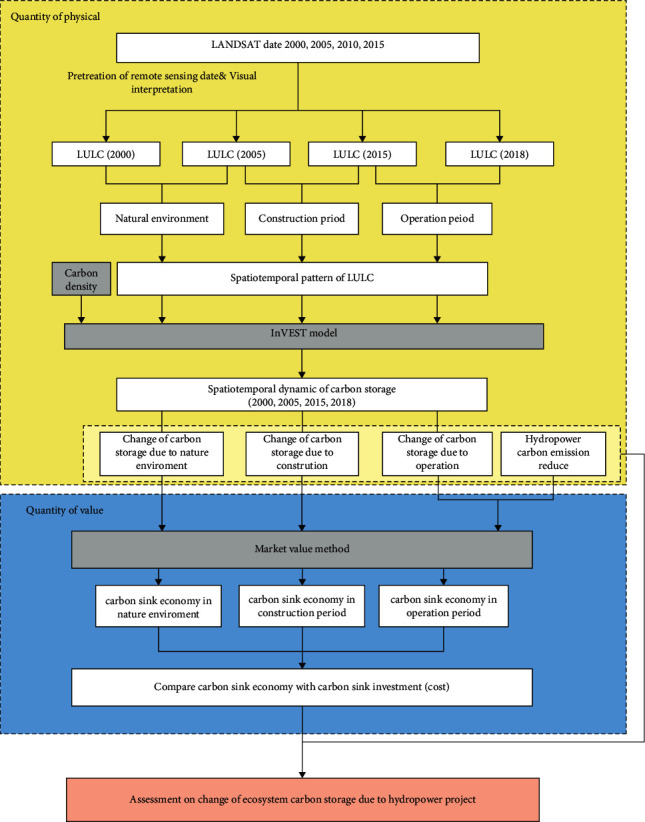
Methodological framework of the study.

The purpose of this study is to provide a framework for assessing impacts of hydropower programs on carbon storage of reservoir ecosystem and provide guidance for future hydropower construction and reservoir ecosystem management. Since the carbon trading market was officially launched in the Shanghai Environment and Energy Exchange (SEEE) in 2021, rights of carbon emission have been endowed with an economic value. Therefore, the evaluation of impacts of hydropower programs on carbon storage can be measured from two aspects: physical quantity and value quantity (Figure 2).

During the studied period from 2000 to 2018, the data obtained from remote sensing image interpretation were used to generate maps of the land use and cover type changes and the land use transfer matrix of the Xiluodu reservoir area from 2000 to 2018. Complementing in-situ monitoring of carbon storage changes, the InVEST model has advantages of data accessibility and visibility to results [25, 26]. It can simulate changes in the value of ecological services of various terrestrial, freshwater, and marine ecosystems. Therefore, the InVEST carbon storage model was used to evaluate physical changes in carbon storage in the reservoir ecosystem caused by hydropower programs.

A change in physical amount of carbon storage in the reservoir area reflects the situation of carbon sequestration and carbon emission. Therefore, a positive net change indicates that the ecosystem absorbs carbon from atmosphere, stores it in the ecosystem, and reduces the content of greenhouse gases in atmosphere. On the other hand, a negative net change indicates that the land use change leads to greenhouse gas emissions. Therefore, considering the carbon emission reduction caused by hydropower programs, the value of the reservoir ecosystem's carbon storage in this study refers to monetizing the carbon sequestration (or carbon emission) and emission reduction by hydropower in the reservoir area. The market value method has been widely used to measure the economic value of the forest carbon sink. Price per unit of the carbon sink is an important factor. This paper uses the market value method to calculate changes in the value of the reservoir ecosystem's carbon storage considering carbon emission trading price of the SEEE. In addition, the calculated value was compared with the carbon sink cost (hydropower investment and operation costs) to discuss the economics of hydropower development.

### 2.3. Calculation Method

#### 2.3.1. Data Source and Processing

We employed the digital elevation model (resolution 30 m × 30 m) to process land use and cover data used obtained from the Data Center for Resources and Environmental Sciences, Chinese Academy of Sciences. The land use and cover data was sourced from Xiluodu reservoir area in 2000, 2005, and 2015, and data in 2018 were from the Landsat TM/ETM+/OLI satellite remote sensing images of the United States Geological Survey (USGS). The remote sensing images were preprocessed by radiometric correction, geometric correction, color enhancement, splicing, cutting, and then artificial visual interpretation. The accuracy of land use and the cover was greater than 85% which meets research requirements.

According to the classification standards issued by the Ministry of Natural Resources of China and the research needs of ecological environment of the Xiluodu reservoir area, the land use and cover types were divided into five types: (1) cultivated land (dry land, paddy land); (2) woodland (woodland, shrubbery, open woodland, and other woodland); (3) grassland (high coverage, medium coverage, and low coverage); (4) water area; and (5) construction land.

#### 2.3.2. Carbon Storage Model: Application of the InVEST Model

In this paper, the carbon storage and capture model in the InVEST model (version 3.5.0) was used to estimate the carbon storage of the reservoir area over time, based on the spatial distribution map of land use at different times and carbon density of each land type. The InVEST model simulated the carbon storage of four carbon pools, including aboveground and underground biomass, dead organic carbon, and soil carbon. The top 20 cm layer of soil was studied in this paper. The calculation formula of carbon storage of reservoir ecosystem is as follows:

(1) $$\begin{array}{c} \mathrm{,,}C_{\text{total}}=C_{\text{above}}+C_{\text{under}}+C_{\text{dead}}+C_{\text{soil}}. \end{array}$$

#### 2.3.3. Carbon Density

The carbon density data of the Xiluodu reservoir area mainly came from areas with a similar natural environment and same classification system as Xiluodu. Data about vegetation type, plant species composition, and vegetation coverage were collected by other scholars via field measurements in the reservoir and nearby areas. Previous studies showed that the conversion coefficient of the biomass to carbon of forest was 0.45–0.50, and that of grassland was 0.40–0.45. The carbon density of the underground root system was determined by ratios of the aboveground biomass to the underground biomass of different land types. The carbon density of the dead organic matter was determined by the ratio of aboveground dead matter to underground dead matter [27, 28]. The final organized data of carbon density of Xiluodu reservoir area are shown in Table 1.

#### 2.3.4. Market Value

Carbon market trading was officially launched on SEEE on July 16, 2021. The closing price on that day was 51.23 CNY/Mg. The carbon in this paper refers to elemental carbon, while the carbon trading in China uses CO_2_eg per ton as trading object. According to the mass conversion coefficient of carbon per ton converted into CO_2_eg, which is 3.67, the carbon trading price per ton in this study is converted to 188 CNY/Mg [40].

A previous study showed that the hydropower carbon footprint of the Xiluodu hydropower station is 7.6 gCO_2_eg/(kW·h), while the coal-power carbon footprint is 822 gCO_2_eg/(kW·h) [41]. The clean energy provided by hydropower station can effectively reduce carbon emissions. By the end of 2018, the cumulative power generation of the station exceeded 300 billion kW·h (date source: China's National Energy Administration, available online), with an emission reduction of 1856.83 million tons of CO_2_eg, equivalent to 50.13 million tons of elemental carbon (C). Therefore, the value of carbon storage in the reservoir area can be calculated as follows:

(2) $$\begin{array}{c} V_{c}=\left(C_{a}+C_{h}\right)\cdot P_{c}. \end{array}$$

*V*_*c*_ is value quantity, *C*_*a*_ is physical quantity change of carbon storage in the reservoir area, *C*_*h*_ is carbon emission reduction by hydropower, and *P*_*c*_ is carbon price (CNY/Mg).

## 3. Results and Analysis

### 3.1. LUCC of the Xiluodu Reservoir Area in 2000–2018

#### 3.1.1. Spatiotemporal Pattern of LULC

**Figure 3:**
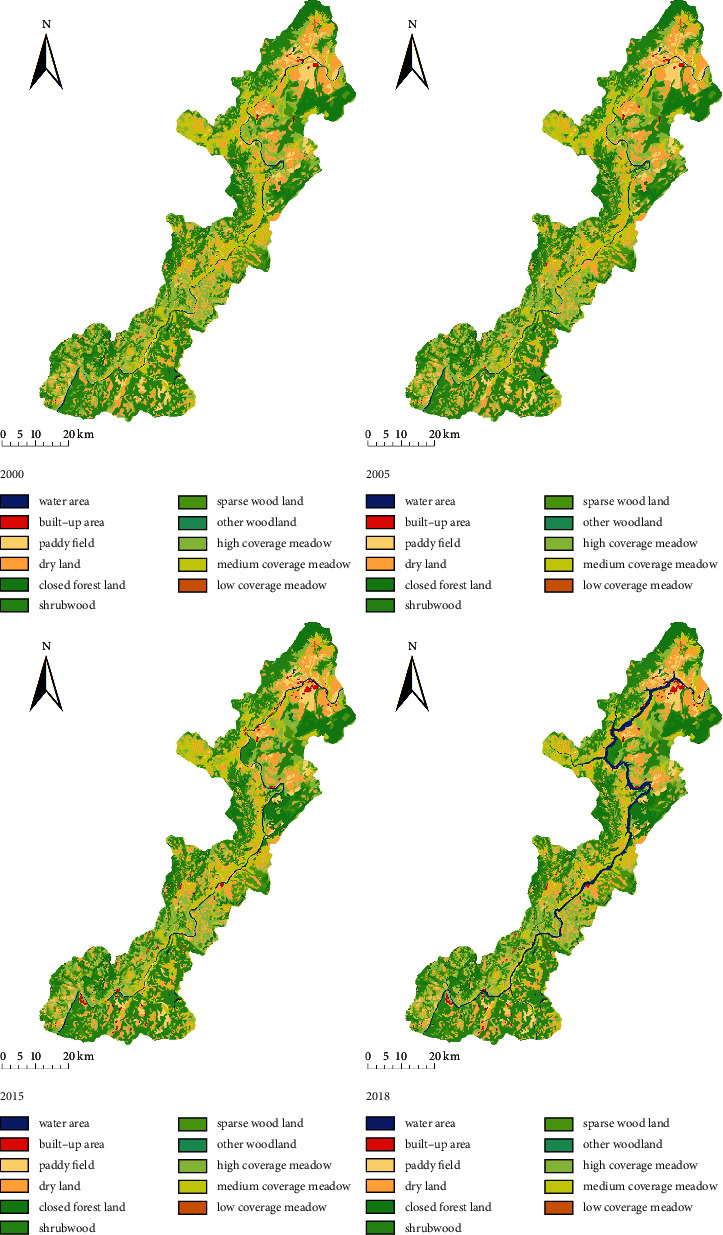
LULC of Xiluodu reservoir area for 2000, 2005, and 2015.

The land use and land cover map (LULC) and area changes in the Xiluodu reservoir area from 2000 to 2018 are shown in Figure 3 and Table 2. Before the start of the construction in 2000, the forestland was the main land use and cover type, accounting for 38.91% of the total area of the reservoir area. It was mainly distributed in the mountainous area with a high elevation and a large slope. The grassland accounted for 32.75% of the total area and was distributed in the middle and low altitude areas along the lower Jinsha River. The water area and the construction land accounted for only 0.9% and 0.19% of the reservoir area, respectively. The water area was distributed in the river valley area with a low altitude level and a gentle slope, while the construction land was scattered in the upstream river.

During the initial stage of the station construction in 2005, no large-scale engineering construction was carried out since it is at the preparation stage of Xiluodu hydropower station. Therefore, the area was kept under the natural environment, and there were no significant changes in the various land use and cover types. After the station's construction in 2015, the area and distribution of the various land use and cover types changed significantly, with an area reduction of the cultivated land and the grassland. The cultivated land area decreased most, reaching 4732.92 hectares. The forestland area, water area, and the construction land area increased to 39.95%, 1.2%, and 0.2%, respectively. The forestland area increased most, reaching 3736.98 hectares. The increase of forestland was mainly on the west bank of Jinsha River in the hinterland, while the increase of construction land was mainly in the low altitude areas at the front and tail of the reservoir. The increase of the water area occurred mainly on both sides of the Jinsha River.

After the station was completed and put into operation in 2018, the cultivated land and grassland areas in the reservoir further decreased. The biggest reduction in the grassland area decreased to 30.77% and was mainly distributed in the middle and low altitude areas of the central region. The forestland and construction land areas decreased by 788.38 hectares and 293.85 hectares, respectively, compared to 2015, and the reduction was mainly distributed on both sides of the Jinsha River. Only the water area increased greatly, and its proportion increased to 3.7%, mainly distributed in the valley area with a low altitude and a gentle slope.

#### 3.1.2. Land Use and Cover Type Change Directly Caused by Hydropower Station Construction

**Figure 4:**
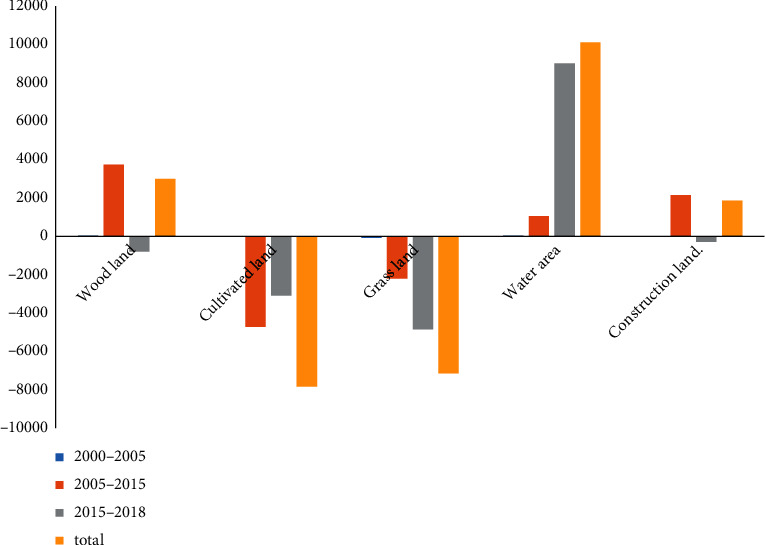
Change in each land use and cover type.

The land use and cover areas of the Xiluodu hydropower station during preparation (2000–2005), construction (2005–2015), and operation (2015–2018) periods are shown in Figure 4. Due to construction, the cultivated land and grassland areas in the reservoir decreased considerably, while the water area increased the most, followed by the forestland and construction land area (Table 3). The following observations are noted:

1. During the construction period, the woodland and construction land were mainly added to the reservoir area, while the cultivated land was mainly removed from the reservoir area. The increased area of both forestland and construction land came from cultivated land and grassland.
2. Change in the construction land was the greatest, which was four times that in 2005. The increased area was mainly the engineering site of station at the head of reservoir and the immigrant resettlement area at the tail of reservoir.
3. The forestland area increased by 3726.98 hm^2^ downstream of the Jinsha River due to the vegetation restoration measures such as afforestation and natural forest closure during events such as “returning farmland to forest,” engineering construction, and immigrant resettlement that began in 1998. Although some forestland was submerged due to water level rise during the station operation, the forestland area still increased compared to 2000. The construction of Xiluodu hydropower station did not cause severe damage to inland plants in the reservoir area.
4. During the operation period, the water level was raised to inundate other lands in the reservoir area, resulting in a significant increase in water area. The cultivated land and grassland were main sources of the increased water area.

### 3.2. Carbon Storage in the Xiluodu Reservoir Area from 2000 to 2018

#### 3.2.1. Spatial and Temporal Distributions of Carbon Storage

**Figure 5:**
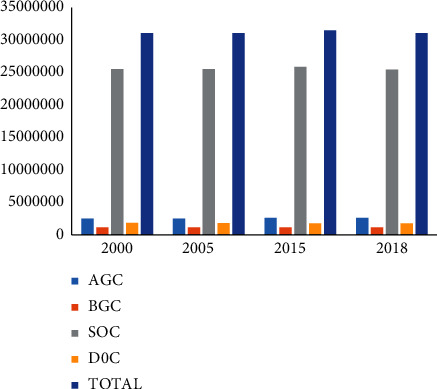
Carbon storage changes of different carbon pools.

The total carbon storage of the Xiluodu reservoir area in 2000, 2005, 2015, and 2018 was 31,057,361 Mg, 31,955,862 Mg, 31,510,555 Mg, and 31,067,232 Mg, respectively (Figure 5). The corresponding average carbon densities were 85.94 Mg/hm^2^, 85.93 Mg/hm^2^, 87.19 Mg/hm^2^, and 85.97 Mg/hm^2^. SOC was the main carbon pool, followed by AGC, DOC, and BGC. The carbon storage of the reservoir ecosystem decreased by 1,499 Mg from 2000 to 2005. The carbon storage increased by 453,193 Mg during the construction period of 2000–2015. The carbon storage decreased by 443,322 Mg during the operation period of 2015–2018. The carbon storage increased by 8504.42 Mg in 2018 compared to 2000 when the hydropower station was not built.

From changes in proportions of land use carbon storage in the reservoir area (Table 4), the forestland was the largest carbon pool in Xiluodu reservoir area. The forestland had a high distribution density and high carbon density, accounting for 54.55%–57.2% of the total carbon storage of the reservoir area ecosystem, and its proportion continued to rise. The carbon storage of the grassland ranked next to the forestland. The grassland and the cultivated land accounted for 25.34%–26.66% and 17.15%–18.73% of the total carbon storage, respectively. The carbon storages of the grassland and the cultivated land continuously decreased each year. The carbon storage of the construction land and the water area ranked the lowest, which accounted for only 0.05%–0.22%. Although the water area increased significantly from 2000 to 2018, its carbon storage was still the lowest which is only 0.02% due to low carbon density.

**Figure 6:**
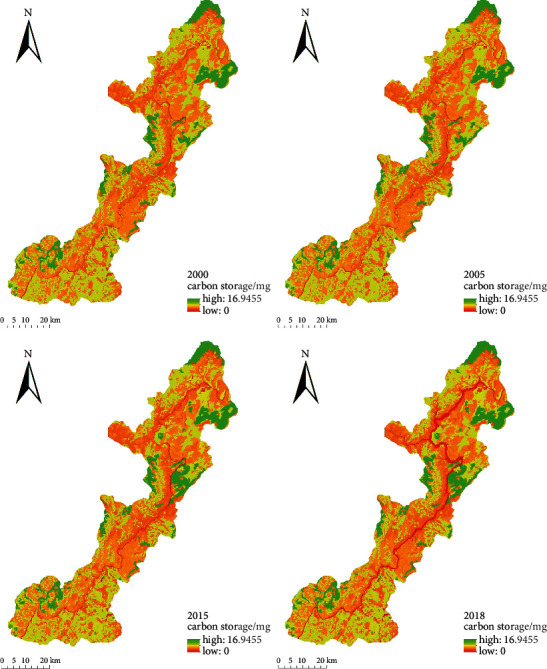
Spatiotemporal changes of carbon storage between 2000 and 2018 (Mg).

In terms of spatial distributions, the carbon storage in the reservoir area was higher in the north and west, but lower in the south and east. The high carbon storage areas were mainly distributed in the high-altitude mountainous forest areas at the upstream and the western boundary of the reservoir. These areas had high vegetation coverage and good vegetation growth which is seldom affected by human activities. Therefore, their natural plants were well maintained. The second high carbon storage area was the grassland area in the south of the reservoir area. The water area was mainly distributed in the mainstream of the Jinsha River and its tributaries with the lowest carbon storage (Figure 6).

**Figure 7:**
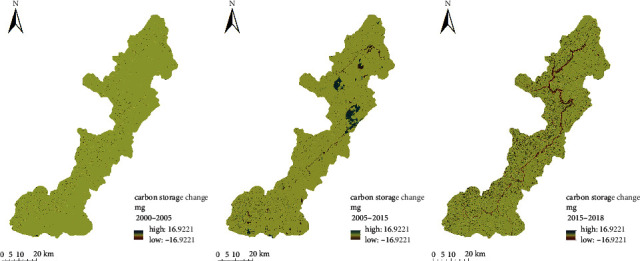
Carbon change between 2000 and 2018.

The carbon storage and spatial distribution did not change significantly from 2000 to 2005. However, with the construction of Xiluodu hydropower station, the carbon storage and spatial distribution changed significantly from 2005 to 2015 during the construction period. The carbon emission was mainly from the hydropower station construction area in the north and the immigrant resettlement area in the south. The carbon sequestration area was mainly the forestland on the western bank of Jinsha River in the middle of the reservoir area. As the station entered its operation period, the carbon storage along the mainstream and tributaries of Jinsha River decreased significantly from 2015 to 2018. Compared with the construction period in 2005–2015, the carbon emission area increased significantly, mainly due to the water level rise in the operation, which flooded vast grassland and cultivated land (Figure 7).

#### 3.2.2. Carbon Storage Change Directly Caused by the Xiluodu Hydropower Station Construction

Due to the construction of Xiluodu hydropower station, the vegetation density, land use, and cover types in the reservoir area changed, which affect the carbon storage of ecosystem (Table 5). From 2000 to 2005, changes in vegetation densities of forestland, cultivated land, and grassland caused carbon densities to change from 120.499 Mg/hm^2^, 59.058 Mg/hm^2^, and 69.982 Mg/hm^2^ to 120.514 Mg/hm^2^, 59.070 Mg/hm^2^, and 70.123 Mg/hm^2^, respectively. The total carbon storage of the unchanged land use and cover types in the reservoir area increased by 5042.82 Mg. With the area conversion to different land use and cover types, the total increase of the carbon storage was 49,746.58 Mg, and the total decrease was 51,244.96 Mg, while the net decrease was 1,498.48 Mg. The annual carbon emissions of the ecosystem were 299.696 Mg/year.

During the construction period from 2005 to 2015, changes in the carbon densities of forestland, cultivated land, and grassland were 123.122 Mg/hm^2^, 58.913 Mg/hm^2^, and 70.123 Mg/hm^2^, respectively. The carbon storage of unchanged land use and cover types increased by 106,863.66 Mg. Due to the area conversion, the total increase of carbon storage was 827,971.28 Mg, and the total decrease was 373,278.88 Mg, while the net increase was 456,892.43 Mg. The annual carbon storage of ecosystem was 45,469.24 Mg/year.

During the operation period from 2015 to 2018, changes in carbon densities of forestland, cultivated land, and grassland were 123.722 Mg/hm^2^, 58.911 Mg/hm^2^, and 70.423 Mg/hm^2^, respectively. The carbon storage of unchanged land use and cover types increased by 96,529.62 Mg. The total increase of the carbon storage caused by changes in land use and cover types was 700,667.40 Mg, and the total decrease was 1,143,990.22 Mg, while the net decrease was 443,322.82 Mg. The annual carbon emissions of ecosystem were 147,774.27 Mg/year.

During the entire study period from 2000 to 2018, the carbon storage of ecosystem caused by land use and cover type change was mainly due to the increase in forest area. The policies of “returning farmland to the forest” downstream of Jinsha River since 1998, the Changsha Shelterbelt Program, and the large-scale artificial planting and natural forest closure management since the hydropower program construction influenced the increase in forest areas. The forest areas converted from other types of land during period of 2000–2005, 2005–2015, and 2015–2018 were 38,644.31 Mg, 607,448.39 Mg, and 309,142.4 Mg, respectively, with ratios of 77.68%, 73.36%, and 44.12%. The carbon increase from 2000 to 2005 mainly came from converting cultivated land to forestland. The carbon increase from 2005 to 2015 mainly came from conversion of cultivated land and grasslands to forestland. The carbon increase from 2015 to 2018 mainly came from the conversion of grassland to forestland.

In these three periods, main sources of carbon loss were different. Before the construction (2000–2005), the carbon loss was mainly due to converting forestland and grassland to other land uses and cover types with a lower carbon density. During the construction (2005–2015) and operation (2015–2018) periods, the carbon losses were mainly due to the conversion of cultivated land and grassland to other land uses and cover types with a lower carbon density.

From 2000 to 2005, the main reason for carbon loss of reservoir ecosystem was the conversion of forestland to cultivated land and grassland. The carbon loss was 38,077.15 Mg, accounting for 74.30% of the carbon loss for that period. This was related to China's strict implementation of cultivated land protection policies, such as “Dynamic Balance of the Total Amount of Cultivated Land in the Region” and “Land Use Control,” and some forestland has been reclaimed for cultivated land. Meanwhile, the regional ecological environment became fragile due to cultivation of steep slopes and reclamation, and some forest vegetation was seriously destroyed and changed into grassland. The construction of Xiluodu hydropower station, immigrant resettlement, construction of supporting facilities, and water storage of the Xiluodu reservoir were important reasons for loss of cultivated land and grassland from 2005 to 2015.

During the construction period (2000–2015), the carbon losses caused by conversion of nonconstruction land and nonwater land into construction land and water area were 99,689.03 Mg and 93,397.37 Mg (corresponding to 26.70% and 25.02%), respectively. During the operation period (2015–2018), the carbon loss caused by conversion from nonwater area to water area was 652,691.21 Mg, accounting for 57.053% of the carbon loss in the same period. On the other hand, the carbon loss caused by conversion of nonconstruction to construction land accounted for 1.25%. It can be observed that, during the construction period, the carbon emission area of the reservoir was distributed in the converted area of nonconstruction to construction land and the submerged area with the rising water level. In contrast, during the operation period, the carbon emission area was only distributed in the submerged area with the rising water level.

### 3.3. Market Value

In order to make the carbon storage value in reservoir area comparable to the cost of the hydropower program from 2005 to 2018, CPI (consumer price index) was used to modify value and cost from 2000 to 2018. The monetary values in this study were prices in 2000. The discount rate of the capital market was assumed to be 8% [28, 42].

In this study, the economic value was evaluated based on the value of carbon storage of reservoir ecosystem during construction (2005–2015) and operation (2015–2018) periods of Xiluodu hydropower station by considering emission reduction. The carbon sink cost refers to the total investment of hydropower station in three periods. From 2005 to 2015, the total static investment of Xiluodu hydropower station was 44.993 billion Yuan in RMB evaluated at the price level of 2005. According to the estimated annual maintenance cost in the environmental assessment impact report of Xiluodu hydropower station, the annual maintenance cost was 1% of the total static investment, i.e., 449 million RMB (2005 price level) [41] (Table 6).

Before the station's construction (2000–2005), there were carbon emissions in the reservoir ecosystem under natural conditions, and the carbon storage value was −0.1 million Yuan in RMB. During the construction period (2005–2015), the carbon storage increased, and the value of carbon storage change was 19 million Yuan. During the operation period (2015–2018), the carbon storage value increased significantly to 611 million Yuan considering the carbon emission reduction caused by hydropower. Moreover, during the construction period (2005–2015), the total investment of the station was 30.621 billion Yuan. We assumed the life of the station was 100 years [41], so the investment was evenly divided to 306 million Yuan per year from the first year of operation. During the operation period (2015–2018), the maintenance cost of the station was 914 million Yuan. With consideration of construction investment, the program cost was 1.828 billion Yuan.

It can be seen that the ecological protection measures during the construction period improved the carbon capture capacity and made a significant contribution to economy of the reservoir area. In addition, the rapid growth of carbon storage value of the reservoir ecosystem was mainly due to hydropower generation. From 2015 to 2018, the value brought by carbon storage was greater than cost on hydropower station's maintenance and slightly exceeded the amortized cost of the hydropower program. Even if the grid electricity price was not considered, the above analysis still demonstrates hydropower development's excellent feasibility and economic value. Furthermore, Xiluodu hydropower station has also played a substantial role in soil erosion prevention, flood control, sand control, and shipping transportation.

## 4. Discussion

Based on the InVEST model and the LULC data obtained from remote sensing images, this paper discusses in detail the physical and economic value changes of carbon storage in Xiluodu reservoir area from 2000 to 2018. The results show that the construction and operation of station generated significant changes in land use, cover types, and fluctuations in ecosystem carbon storage in the reservoir area. The hydropower program made positive contributions to economy of the reservoir area:

1. The construction of hydropower station disturbed the reservoir ecosystem. However, after the construction completion, the ecological restoration measures brought the carbon storage level of the reservoir ecosystem higher than the preconstruction level even though a large amount of land was submerged in the water area. With the comparison of status of before and after station construction, it was found that the forestland, water area, and construction land in the reservoir area increased, while the areas of cultivated land and grassland decreased. The water area reached 10,118.07 hectares which increased most because of water storage in the reservoir from 2015 to 2018. The water has inundated cultivated land and grassland along the Jinsha River and its tributaries. The cultivated land had the largest reduction in area, reaching 7,835.13 hectares, mainly due to water flooding, immigrant resettlement, land occupation for the station construction, and ecological conversion of farmland.
2. In terms of physical quantity, the carbon storage in Xiluodu reservoir area increased by 8,504.42 Mg from 2000 to 2018. The spatial distribution of carbon storage exhibited a trend of higher in the north and west while lower in the south and west. During the construction period (2005–2015), the station occupied a large amount of nonconstruction land, and the water level rose to flood nonwater area resulting in the loss of carbon storage. Still, the forest area increased compared to 2005 due to implementation of water and soil conservation and vegetation restoration policies which increased the carbon storage of ecosystem. Considering all land use and cover area, the carbon storage of reservoir ecosystem increased during the construction period. During the operation period (2015–2018), the reservoir water level rose and flooded large cultivated land and grassland. This was the main reason for the loss of carbon storage of ecosystem. However, compared to 2000 when the construction was not started yet, the carbon storage in 2018 was more significant. The change of carbon storage mainly comes from the losses of carbon storage in the construction area in the north of the reservoir area, the resettlement area in the south, the main stream and tributaries of the lower Jinsha River, and the increase of carbon storage in the central and western bank of the Jinsha River. This change may be due to increased vegetation coverage, reduced soil erosion, and a stable terrestrial ecological environment after the construction of station.
3. During the operation period (2015–2018), the value change of carbon storage of the reservoir ecosystem was greater than operation and maintenance cost on hydropower station and exceeded the station's amortized cost. Even if the income from grid electricity price was not taken into account, the analysis still showed that hydropower development is feasible and economical.
4. Coal power (49.07%, from the year 2020 data) has been the largest energy source in China. It has become the main option of electrical power development due to low construction cost and fast production. However, the operation cost has increased and the profits have decreased with emergence of carbon emission. Although the initial investment cost of hydropower is higher and the construction period is longer, its sales income of carbon emission rights in the operation period is greater than the maintenance cost and even the investment cost. Therefore, the economic value of hydropower is significantly higher than that of coal power.

## 5. Conclusions

1. This study proposes a framework for assessing impacts of hydropower programs on the reservoir ecosystem's carbon storage. It provides recommendations for future land management in the reservoir area and hydropower development management in Jinsha River Basin and other areas. The case study of Xiluodu reservoir area found that afforestation and forest restoration are main factors leading to the increase of carbon storage in reservoir area. Other effective measures to increase carbon storage of the reservoir system include a reasonable plan of the proportion of green and construction land in future land management; conversion from cultivated land to forest land; and increase of vegetation coverage and quality. Moreover, vegetation restoration measures such as natural forest closure management and afforestation in future hydropower development can effectively mitigate the disturbance to the reservoir ecosystem. The land use and cover type with a low carbon density should be selected as much as possible when determining the inundated area to reduce carbon loss caused by the rising water level in the reservoir. In addition, evaluating the carbon storage value of the reservoir ecosystem can help in decision-making of hydropower development.
2. This paper has several limitations that are subject to future study. First, most carbon density data came from previous literature and were fixed values with which their time variations were disregarded. The constant assumption is different from real situation since the carbon densities are more complex in different land uses and cover types in different periods. Therefore, dynamic carbon densities need to be obtained in future through detailed field monitoring in the reservoir area to improve the accuracy of results. Secondly, the InVEST model can also be used to quantify the ecosystem services in the reservoir area in addition to carbon storage assessment, such as habitat quality and soil erosion. The impacts of hydropower programs on various ecological services in the reservoir area can be assessed in future studies.

## Figures and Tables

**Table 1:** Carbon densities of different land use types in Xiluodu reservoir area.

Land use and cover types (Mg/hm^2^)		*C*_*i*,above_	*C*_*i*,under_	*C*_*i*,soil_	*C*_*i*,dead_	Reference
Category	Type					
Woodland	Woodland	31.95	6.38	146.82	2.96	[29–31]
	Shrubwood	8.1	1.62	91.7	2.48	[31, 32]
	Open woodland	8.1	1.62	91.7	2.48	[29, 30, 33]
	Other woodland	35.03	7.01	142.58	3.75	[29, 30, 33]

Cultivated land	Paddy field	5.42	1.96	92.9	0	[34, 35]
	Dry land	3.64	0	33.46	13	[35, 36]

Grassland	High coverage	2.75	7.37	64.03	4.07	[37, 38]
	Medium coverage	2.205	5.365	48.41	3.035	[37, 38]
	Low coverage	1.66	3.36	25.79	2	[37, 38]

Water area	Water area	0.3	0	0	0	[39]

Construction land	Construction land	0	0	22.25	0	[39]

**Table 2:** Land use and cover change between 2000 and 2018.

Land use type	2000		2005		2015		2018	
	Area (hm^2^)	Proportion (%)	Area (hm^2^)	Proportion (%)	Area (hm^2^)	Proportion (%)	Area (hm^2^)	Proportion (%)
Woodland	140601.51	38.91	140651.28	38.92	144388.26	39.95	143599.88	39.73
Cultivated land	98495.55	27.26	98483.31	27.25	93750.39	25.94	90660.42	25.09
Grassland	118334.79	32.75	118250.82	32.72	116041.59	32.11	111189.33	30.77
Water area	3246.75	0.90	3287.25	0.91	4340.97	1.20	13364.82	3.70
Construction land	702.45	0.19	707.13	0.20	2859.93	0.79	2566.08	0.71

**Table 3:** The land use transfer matrix of the Xiluodu reservoir area from 2000 to 2018.

Time	Land use type	Woodland	Cultivated land	Grassland	Water area (hm^2^)	Construction land
2000–2005	Woodland	139961.25	313.02	313.83	11.97	1.08
	Cultivated land	388.89	97684.92	389.34	23.13	9.09
	Grassland	292.59	472.77	117530.82	37.8	0.72
	Water area	7.2	8.37	16.02	3214.35	0.18
	Construction land	1.35	4.23	0.81	0	696.06

2005–2015	Woodland	137507	1101.69	1685	217.80	139.77
	Cultivated land	2513	91321.56	2606	467.73	1574.37
	Grassland	4265	1195.02	111614	661.05	516.15
	Water area	38	120.06	135	2993.58	0.81
	Construction land	65	11.88	1	0.36	628.83

2015–2018	Woodland	135881.64	3437.82	3821.22	1211.76	35.46
	Cultivated land	3532.86	82451.25	4587.84	2950.29	227.88
	Grassland	4086.55	4578.12	102558.87	4759.11	59.49
	Water area	66.16	40.5	50.76	4179.33	3.69
	Construction land	32.67	152.73	170.64	264.33	2239.56

**Table 4:** Carbon storage of different land use and cover between 2000 and 2018.

Land use type	2000	2005	2015	2018
	Proportion (%)	Proportion (%)	Proportion (%)	Proportion (%)
Woodland	54.55	54.56	56.42	57.20
Cultivated land	18.73	18.73	17.56	17.15
Grassland	26.66	25.65	25.83	25.34
Water area	0.00	0.00	0.00	0.02
Construction land	0.05	0.05	0.20	0.20

**Table 5:** Changes of carbon storage in the process of land use change from 2000 to 2018.

Time	Land use type	Woodland	Cultivated land	Grassland	Water area (Mg)	Construction land
2000–2005	Woodland	2193.84	−19228.63	−18848.52	−1638.30	−104.67
	Cultivated land	22879.36	1134.07	4259.64	−1358.15	−322.54
	Grassland	14769.21	−7078.17	1680.14	−2632.57	−33.41
	Water area	864.88	491.57	1115.94	0.00	4.18
	Construction land	130.86	150.35	37.64	0.00	34.80

2005–2015	Woodland	160962.94	−14313.2	28801.23	−27469.83	−55890.3
	Cultivated land	366742.35	−67807.95	−84823.58	−26162.65	−13542.65
	Grassland	227569.74	−13247.75	13708.67	−46048.19	−23964.42
	Water area	5665.72	7032.31	9420.57	0.00	18.81
	Construction land	7470.58	531.82	46.54	−8.36	0.00

2015–2018	Woodland	228882.08	−113.63	55349.34	−172808.43	−8054.04
	Cultivated land	78510.50	−220115.17	−199255.54	−148782.57	−3530.12
	Grassland	219199.17	−51320.75	87762.710	−331100.21	−2769.378
	Water area	8161.49	2372.16	3585.571	0.00	85.71
	Construction land	3271.24	5397.78	8089.65	−6140.38	0.00

**Table 6:** Carbon storage value and carbon sink cost evaluated at the 2000 price level.

Economic benefit and cost	2000–2005	2005–2015	2015–2018
Value (100 million RMB)	−0.001	0.19	19.87
Hydropower program cost (100 million RMB)	0	306.21	9.14
Amortized cost of hydropower program (100 million RMB)	0	0	18.28

## Data Availability

The data used to support the findings of this study are included within the article. The original details of the data presented in this study are available on request from the corresponding author.
